# Impact of Olive Oil Supplement Intake on Dendritic Cell Maturation after Strenuous Physical Exercise: A Preliminary Study

**DOI:** 10.3390/ijerph18084128

**Published:** 2021-04-14

**Authors:** Laura Esquius, Casimiro Javierre, Inés Llaudó, Inés Rama, Guillermo R. Oviedo, Marta Massip-Salcedo, Alicia Aguilar-Martínez, Oscar Niño, Núria Lloberas

**Affiliations:** 1 Department of Physiological Sciences, Medical School, University of Barcelona, 08007 Barcelona, Spain; lesquius@uoc.edu; 2 Foodlab Research Group, Faculty of Health Sciences, Universitat Oberta de Catalunya, 08018 Barcelona, Spain; mmassips@uoc.edu (M.M.-S.); aaguilarmart@uoc.edu (A.A.-M.); 3 Nephrology Department, Bellvitge University Hospital, IDIBELL, 08907 Barcelona, Spain; illaudo@gmail.com (I.L.); irama@bellvitgehospital.cat (I.R.); nlloberas@ub.edu (N.L.); 4 Faculty of Psychology, Education and Sport Science-Blanquerna, University Ramon Llull, 08022 Barcelona, Spain; guillermorubeno@blanquerna.url.edu; 5 Faculty of Sports Sciences and Physical Education, University of Cundinamarca, Cundinamarca 252212, Colombia; oscarnio@gmail.com

**Keywords:** olive oil, physical exercise, inflammation, dendritic cells

## Abstract

Physical exercise is known to have a dose-dependent effect on the immune system and can result in an inflammatory process in athletes that is proportional to the intensity and duration of exertion. This inflammatory process can be measured by cell markers such as dendritic cells (DCs), which, in humans, consist of the myeloid DC (mDCs) and plasmacytoid DC (pDCs) subpopulations. The aim of this study was to measure DC differentiation to determine the possible anti-inflammatory effects, after intense aerobic effort, of the intake of a 25 mL extra-virgin olive oil supplement. Three healthy sports-trained subjects went through resistance exercise loads on two days separated by a week: on one day after active supplement intake and on the other day after placebo supplement intake. The results show that the highest increase (77%) in the percentage of mDCs as a proportion of pDCs was immediately after testing. Independently of the supplement taken, mature mDCs showed a decreasing trend between the test one hour after and 24 h after testing ended. Nevertheless, measured in terms of the coefficient of variation, only the decrease (46%) for extra-virgin olive oil supplementation was statistically significant (95% CI: 30–62%; *p* = 0.05). In conclusion, an extra-virgin olive oil supplement could reduce the inflammatory impact of intense aerobic effort and improve recovery at 24 h.

## 1. Introduction

Olive oil is the main cornerstone of the Mediterranean diet and is the most important differentiating factor compared to other countries as a source of polyphenols [1]. Olive oil consumption in Mediterranean countries is estimated between 30–50 g·day^−1^. A daily consumption of 50 g of olive oil with an average phenol concentration of 180 mg·kg^−1^ would result in an estimated consumption of approximately 9 mg·day^−1^ [2].

In Mediterranean countries, olive oil has always been considered beneficial for health, and this assumption has been confirmed by the results of numerous studies. Olive oil consumption, specifically the extra-virgin variety, is associated with a reduced inflammation, cardiovascular disease, cancer, and mortality [2,3,4,5,6,7,8]. These benefits may be related to its source of polyphenols, which have been shown to possess antimicrobial, antioxidant, and anti-inflammatory systemic properties [6,9,10,11,12].

Physical exercise can have both positive and negative effects on immune system depending on its duration and intensity [13,14,15]. Previous research has shown that during prolonged physical exercise cortisol release from the adrenal cortex predominates, resulting in modulation of cytokine secretion, alteration of circulating white blood cells, and efficacy of antigen presentation.

Dendritic cells (DCs), which are derived from bone marrow progenitor cells, are potent antigen-presenting cells that play a major role in initiating and maintaining innate and adaptive immunity. DCs form a dense network in nearly all body tissues [16], and can be found in an immature state as DC precursors in the blood. Their main function is to detect potentially harmful foreign antigens in the internal environment [17]. As a consequence of antigen uptake and the presence of inflammatory stimuli, immature DCs undergo terminal differentiation.

Humans have two different blood circulating DCs subpopulations: myeloid DCs (mDCs) and plasmacytoid DCs (pDCs). The mDCs, similar to monocytes, can be further divided into the more common myeloid type 1 DCs (mDC1), a major stimulator of T-cells, and the extremely rare myeloid type 2 DCs (mDC2s), which may have a function in fighting wound infection. The pDCs, which look like plasma cells, are often referred to as interferon-producing cells but have certain characteristics similar to mDCs [18].

Several studies have explored the effects of acute bouts of exercise on DC differentiation [19,20,21,22,23,24,25]. When exercise intensity remains constant, the pattern of substrate utilization changes. Hence, the longer the time spent exercising, the higher the contribution of fat as an energy substrate. In prolonged exercise, as glycogen stored in working muscles is depleted, plasma fatty-acid oxidation increases, due to an increase in circulating catecholamines (adrenalin and noradrenalin) and a decrease in circulating insulin. The catecholamines and insulin play specific roles in stimulating and inhibiting, respectively, the lipolysis process.

Compared to untrained athletes, those trained in endurance sports use less muscle glycogen and have a greater capacity to use fatty acids as an energy source, which explains why trained athletes perform better in endurance events [26,27]. Thus, fat supplementation could serve as a nutritional strategy to reduce muscle glycogen depletion in endurance exercise, since the rate of oxidation of free fatty acids in muscle depends in part on their concentration levels in blood plasma [28].

Extra-virgin olive oil is a source of fatty acids that is rich in polyphenols [1]. A study assessing the beneficial effects of 25 mL dose of olive oil found that it neither induces significant postprandial lipaemia nor increases markers of oxidation in-vivo [29]. To the best of our knowledge, there is a gap in the literature regarding the effects of the use of extra-virgin olive oil in endurance sports. Therefore, the main objective of this study was to evaluate the anti-inflammatory effects of an acute fat supplementation rich in a natural anti-inflammatory substance—extra-virgin olive oil with a high polyphenol content—in individuals performing high-intensity exercise with an energy cost equivalent to 2000 kcal.

## 2. Materials and Methods

### 2.1. Participants

The participants volunteering in the study were 3 physically active men who perform recreational sports training 3 to 5 days a week. Informed consent was obtained in each case, and the study protocol, compliant with the principles of the Declaration of Helsinki, was approved by the Institutional Ethics Committee.

### 2.2. Study Design

In this randomized cross-over controlled double-blind trial, two separate effort test sessions were carried out separated by one week. No physical activity was performed the day before testing. Supplementation in the form of a gel at a temperature of about 10 °C was orally administered blind to each of the three subjects: an active supplement with extra-virgin olive oil or a placebo supplement without extra-virgin olive oil. In what follows, the subjects who received the supplement with olive oil and the placebo are referred to as treated and non-treated subjects, respectively. The order of supplementation was randomized using a random number generator (i.e., day 1 = treated/day 2 = non-treated or day 1 = non-treated/day 2 = treated).

### 2.3. Supplements

The active and placebo supplements were prepared—in the same laboratory of the Department of Physiological Sciences (University of Barcelona)—using orange juice, with added modified starch to achieve a gel texture. The active supplement also included extra-virgin olive oil (Table 1). The calorific value and fat content of the active supplement were much higher than in the placebo.

### 2.4. Protocol

Effort testing was performed in the laboratory of the Department of Physiological Sciences (University of Barcelona) at an ambient temperature of 22–24 °C and with relative humidity at 55–65%.

A venous catheter was placed in a superficial forearm vein to facilitate the collection of blood samples at five different times. On each occasion, 2–3 mL of peripheral venous blood was extracted into a tube to determine the following: monocyte subpopulations, DC phenotype, and lactate concentration (lithium heparin tubes); biochemical profile (Serum Sep Clot Activator tubes); and complete blood count (K3EDTA tubes).

**Figure 1:**
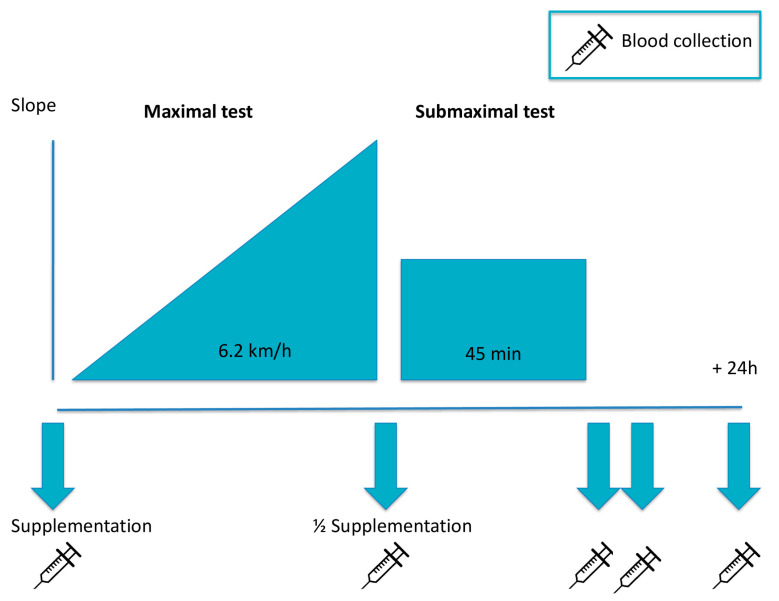
Protocol stages.

The protocol stages were as follows (see also Figure 1):

**Step 1. Supplement 1**. Blood was sampled one hour before the test (T0) and the supplement (active or placebo, depending on the day) was administered.

**Step 2. Maximal test**. Participants performed a maximal incremental exercise test starting at 6.2 km/h for 4 min, after which grade increased by 1% every 4 min, up to exhaustion point. Blood was sampled immediately after the end of the test (T1).

**Step 3. Supplement 2**. Recovery time of 5 min. A half supplement (active or placebo, depending on the day) was administered with 500 mL of water.

**Step 4. Submaximal test**. Maximum distance (speed controlled by the subject) covered in 45 min, with slope fixed at 50% of peak (e.g., 10% if 20% of the maximum slope was reached in the maximal test). Blood was sampled immediately after (T2) and one hour after (T3) this test.

**Step 5. Follow-up.** Blood was sampled 24 h after the test (T4).

### 2.5. Dietary Assessment

The volunteers consumed a specific diet, based on standard energy requirements, the day before testing and at breakfast on the day of testing to ensure no nutritional variability in macronutrient intake of antioxidants, especially phenolic compounds. The diet, designed using the EasyDiet online software, was based on approximately 3000 kcal/day (60% carbohydrates, 25% fat, and 15% proteins).

### 2.6. Analytical Procedures

#### 2.6.1. Physical Test and Metabolic Analysis

A treadmill was used as the ergometer (Quasar, HP Cosmos Sports and Medical Gmbh, Nussdorf-Traunstein, Germany). Electrocardiographic (ECG) parameters were measured using specific software (CardioScan v.4.0, DM Software, Stateline, NV, USA). Arm blood pressure (BP) was taken manually using a clinical sphygmomanometer (Erkameter 3000, Erka, Bad Tölz, Germany). O_2_ uptake and CO_2_ production were measured by an automatic gas analysis system (Metasys TR-plus, Brainware SA, La Valette, France) equipped with a pneumotach and a two-way mask (Hans Rudolph, Shawnee, KS, USA). Gas and volume calibrations were performed before each test according to manufacturer guidelines.

#### 2.6.2. Antibodies and Reagents

The following monoclonal antibodies were obtained from Invitrogen (Molecular Probes, Eugene, OR, USA): CD14 TRI-COLOR conjugate and human CD16-fluorescein isothiocyanate (FITC). Fluorescence-activated cell sorting (FACS) lysing solution and CellFIX were purchased from Becton Dickinson Pharmingen (San Diego, CA, USA), along with other monoclonal antibodies: anti-human CD40-FITC, CD80-FITC, CD83-FITC, CD86-FITC, and human leukocyte antigen D-related (HLA-DR) phycoerythrin (PE). Human Hematopoietic Lineage APC Cocktail, anti-human CD123-FITC, and CD11c-PE-Cy5 were obtained from e-Bioscience (San Diego, CA, USA). Finally, the Cytometric Bead Array (CBA) Human Inflammatory Cytokines Kit was obtained from BD Biosciences (San Diego, CA, USA).

#### 2.6.3. Flow Cytometry Analysis of DC Phenotype, Co-Stimulatory Molecules, and Subsets

For DC identification, peripheral blood was stained by Human Hematopoietic Lineage APC Cocktail, anti-human CD123-FITC and CD11c-PE-Cy5, anti-human CD40-FITC, CD80-FITC, CD83-FITC, CD86-FITC, and HLA-DR PE. Whole blood (100 µL) was incubated with different combinations of antibodies for 20 min in darkness at room temperature. Red blood cells were lysed in 2 mL of FACS lysing solution for 10 min at room temperature. Samples were immediately washed twice with phosphate buffered saline (PBS), re-suspended with 250 µL of CellFIX and stored at 4 °C until the flow cytometry analysis. FACS was performed using FACS Canto and analyzed with FACS Diva software (Becton Dickinson Pharmingen). DCs were defined as Lineage^−^/HLA-DR^+^, the myeloid subset (mDCs) as CD11c^+^/CD123^−^ and the plasmacytoid subset (pDCs) as CD11c^−^/CD123^+^. In both DC subsets, different co-stimulatory molecule expression (CD80, CD40, CD83, and CD86) were used to define their maturation state.

#### 2.6.4. Flow Cytometry Analysis of Monocyte Subpopulations in Peripheral Blood

**Figure 2:**
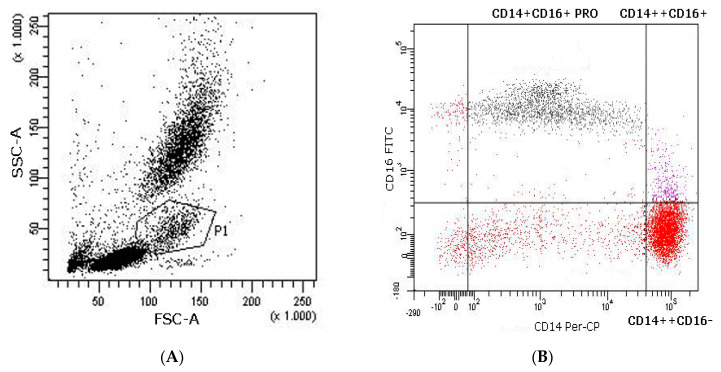
Representative flow cytometry analysis of monocyte subsets in the peripheral blood of a volunteer after an exercise bout. (**A**). Monocytes gated in P1 in FCS-A/SSC-A. (**B**). Monocyte subsets in P1 assessed using a CD14 Per-CP/CD16-FITC dot plot.

To identify the monocyte subpopulations, whole blood (100 µL) was incubated with human CD14 TRI-COLOR conjugate and human CD16-FITC for 20 min in darkness at room temperature. Red blood cells were lysed, using 2 mL of FACS lysing solution, for 10 min at room temperature, after which samples were immediately washed twice with PBS, re-suspended with 250 µL of CellFIX and stored at 4 °C until flow cytometry analysis. Figure 2 shows a representative distribution of the monocyte subpopulations as measured by flow cytometry in the peripheral blood of a volunteer after an exercise bout. The monocyte population (P1) was defined for forward scatter FSC-A and side scatter SSC-A. Monocyte subsets within the P1 population were subsequently assessed using a CD14 Per-CP/CD16-FITC dot plot.

#### 2.6.5. Flow Cytometry Cell Cytokine Analysis

Interleukin (IL)-1β, -6, -8, -10, -12p70, and tumor necrosis factor (TNF)-α secreted protein levels from the plasma were measured quantitatively using the CBA Human Inflammatory Cytokines Kit. Flow cytometry analysis was performed using FACS Canto and cytokines’ concentrations were analyzed by FCAP Array™ software. Cytokines were quantified for each subject’s plasma at each monitoring point. The main results are shown in supplementary material.

### 2.7. Statistical Analysis

Each analysis was performed three times and representative data were compiled. The non-parametric Friedman test for repeated measures was used to evaluate mDC and pDC changes between the three analyses. Statistical analyses were conducted using Statistical Package for the Social Sciences (SPSS) v.14.0 (IBM SPSS Statistics, Chicago, IL, USA). Statistical significance was set at an alpha level < 0.05.

## 3. Results

### 3.1. Participants

Three healthy, physically active male volunteers were selected to participate in the study by means of non-probabilistic intentional sampling. The volunteers were aged between 35 and 51 years, with height between 165 and 181 cm, weight between 65 and 94 kg, and body mass index (BMI) between 22.39 and 28.75 (Table 2).

### 3.2. Analysis of pDC and mDC Populations and Monocytes after Exercise

**Figure 3:**
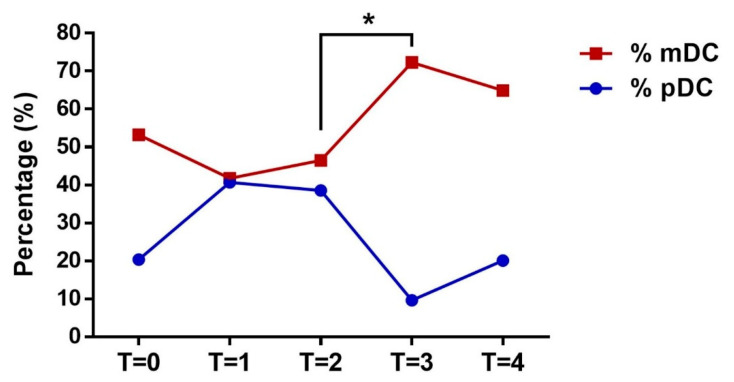
Analysis of mDC (red line) and plasmacytoid DC (pDC) (blue line) subtypes at different monitoring points (T0 to T4) according to the specific phenotypic markers Lin^−^ HLADR+ CD11+/− CD123−/+. * Shows a strong maturation response observed for mDCs that mirrored by a decrease in pDCs.

**Figure 4:**
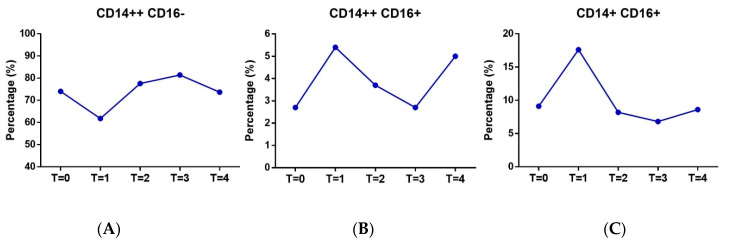
Molecule expression in monocytes analyzed at different monitoring points (T0 to T4), showing percentage of monocyte subsets before, during, and after testing. (**A**). CD14++CD16− (**B**). CD14++CD16+ (**C**). Pro-inflammatory CD14LowCD16+.

DC populations were analyzed by FACS to determine mature forms in the peripheral blood of the subjects according to forward-/side-scatter profiles and specific maturation markers established in a previous study [30], namely CD40, CD80, CD83, CD86, and HLA-DR. Mature DCs were differentiated as mDC and pDC subpopulations, depending on maturation subtype according to the specific phenotypic markers Lin^−^ HLADR^+^ CD11+/− CD123−/+ at the different monitoring points (T0, T1, T2, T3, and T4) (Figure 3). At T3, a strong maturation response was observed for mDCs (*p* < 0.05) that was mirrored by a decrease in pDCs. Monocytes were also gated from blood samples and analyzed by FACS (CD14+/++ CD16+/−) to differentiate pro-inflammatory from transitional monocytes (Figure 4). Results show an increase in pro-inflammatory monocytes during exercise with no variation in cardiovascular risk-related monocytes.

### 3.3. Impact of Olive Oil Supplement on DC Maturation

**Figure 5:**
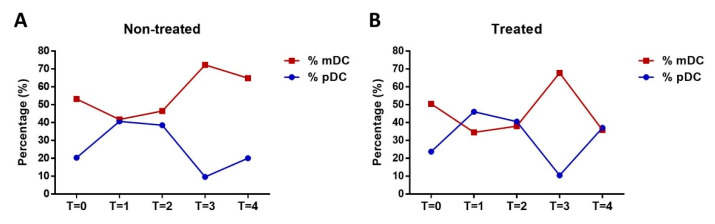
Impact of olive oil supplement on mature dendritic cells (DCs) (mDC: red line; pDC: blue line) during and after intense exercise ((**A**). Non-treated group and (**B**). Treated group). The percentage of myeloid DCs (mDCs) increased in both treated and non-treated subjects from T2 to T3. The decrease in mDCs at T4 (24 h after the exercise) was greater for the treated subjects.

To evaluate olive oil supplement in DC maturation after intense physical exercise, mDCs and pDCs in the treated and non-treated subjects were identified at the different monitoring points. Mature DCs as a proportion of mDCs increased most rapidly from T3 and then declined towards T4 (Figure 5). Nevertheless, this decrease (∆T3-T4/∆T3-T2) measured in terms of the coefficient of variation (CV) was only significant for treated subjects (95% CI: 30–62%, *p* = 0.05) (Table 3). All mean values were analyzed in three repetitions of the analysis.

### 3.4. Metabolic Data

Metabolic data results were not statistically significant (Table 4).

## 4. Discussion

Physical exercise is known to have a dose-dependent effect on the immune system. While regular moderate exercise has the potential to enhance immune functions, excessive and prolonged exercise may induce immunosuppression [31].

Regular moderate exercise is considered to produce long-term anti-inflammatory effects [32]. In contrast, strenuous exercise has been shown to induce rapid systemic cytokine release and to activate neutrophils and monocytes [33,34]. This inflammatory response seems to be balanced by an enhanced concomitant release of anti-inflammatory defenses such as interleukin 10 (IL-10). A balance between pro- and anti-inflammatory cytokines has been argued to prevent or restrict exercise-induced oxidative stress [33].

In the present study, we analyzed the impact of strenuous physical exercise on the number and maturation of peripheral blood DC subsets and on metabolic data and compared the results for the same men when they had received a supplement with extra-virgin olive oil rich in polyphenols and when they had received a placebo.

The results show that, although metabolic effort was similar, the anti-inflammatory response was different, with the findings of the present study being, therefore, consistent. In parallel, the different parameters show a response for this type of effort that is both intense and long-lasting. Observed was a response that may be related to a reduced nonspecific immune response, although without statistical or clinical significance.

The impact of physical exercise on the characteristics of mDCs and pDCs has not as yet been described, although previous studies have examined and determined the acute effect of exercise on DC differentiation [19,20,21,22,23,24,25].

In our study, the impact of physical exercise on mDC and pDC characteristics seems to be congruent, given that all participants responded in the same direction but with important differences, in that the percentage of mDCs increased in both groups. Suchánek et al. [21] analyzed the frequency and absolute numbers of circulating myeloid and plasmacytoid DCs in peripheral blood and evaluated their maturation status before and after the physical load in 18 profesional ice-hockey players. However, compared to our results they observed that both myeloid and plasmacytoid DCs increased significantly.

The results show that the highest increase (77%) as a proportion of mDCs was immediately after testing, whereas DC maturation showed a decreasing tendency from immediately after exercise to 24 h after exercise for all individuals, independent of the supplement taken. Nevertheless, measured in terms of the CV, it was only when the subjects had received extra-virgin olive oil that this decrease (of 46%) proved to be statistically significant, suggesting that the polyphenol content in the oil affected response. Lackermair et al. examining the effect of stress on dendritic cell count in a cohort of 100 healthy athletic males and the influence of a polyphenol-rich beverage [35]. They evaluated both moderate exercise during a 4-week training interval and the strenuous exercise of a marathon run, 24 h and 72 h after. Marathon running induced a significant increase of circulating mDCs and a significant decrease of pDCs. However, compared to our results, polyphenol supplementation did not significantly affect mobilization of dendritic cells.

Bowtell et al. carried out a systematic review that evaluated the effect of fruit-derived polyphenol supplementation for athlete recovery and performance. Evidence would suggest that acute supplementation with ~300 mg polyphenols 1–2 h prior to exercise may enhance exercise capacity and/or performance during endurance exercise via antioxidant and vascular mechanisms. Additionally, supplementation with >1000 mg polyphenols per day for 3 or more days prior to and following exercise will enhance recovery following muscle damage via antioxidant and anti-inflammatory mechanisms [36].

Polyphenol supplementation in exercise studies included mainly extracts (multicomponent or purified), juices, infusions, or an increased intake of polyphenol-rich foods [37]. In our study, for the extra-virgin olive oil supplement, we did not quantify the polyphenols in either the olive oil or in the blood. However, using this kind of inflammation marker, we can assess the impact of inflammation before, during and 24 h after effort, it was observed that an extra-virgin oil supplement results in better recovery and an improved inflammatory state.

Our findings should be discussed in the light of our methodological limitations. First, the small number of participants and the fact that we only recruited men for this study. The complexity of the protocol, however, needed motivated volunteers with time availability. Studies with larger samples would be necessary to confirm our findings, explore individual differences in responses, and compare sex differences. Second, we did not use biomarkers to observe physiological oxidative status. However, it has been demonstrated that a 25-mL dose of extra-virgin olive oil does not induce significant postprandial lipaemia nor does it increase in vivo oxidation markers [29]. Further research should be conducted to provide an accurate picture of the effectiveness of extra-virgin olive oil supplement on dendritic cell maturation after strenuous physical exercise.

## 5. Conclusions

Strenuous exercise that generates fatigue to exhaustion has short-term proinflammatory implications that decrease immune capacity. The intake of extra-virgin olive oil with its high polyphenol content seems to reverse the inflammatory state to normal levels of mDC within 24 h. The data observed in this experiment are an objective basis that allows exploring the possibilities of DCs as an inflammatory marker against different types of efforts and evaluating the protective effect of certain supplements.

## Figures and Tables

**Table 1:** Supplement ingredients and nutritional content.

	Active Supplement	Placebo Supplement
Ingredients	100 mL commercial orange juice 8 g modified starch 25 mL extra-virgin olive oil	100 mL commercial orange juice 8 g modified starch
Energy (Kcal)	277	52.8
Fat (g)	25	0.1
Carbohydrates (g)	12.6	12.6

**Table 2:** Subject characteristics.

Subject	#1	#2	#3
Age (years)	51	34	39
Weight (kg)	94	65	71
Height (cm)	181	165	178
BMI (kg·m^−2^)	28.75	23.90	22.39

**Table 3:** Coefficient of variation (∆T3-T4/∆T3-T2) evaluating recovery from exercise-induced inflammation in treated and non-treated subjects.

Subjects	Non-Treated			Treated		
	T2	T3	T4	T2	T3	T4
#1	56.4	57.8	56	62.6	77.9	37.1
CV1	1.29			2.63		
#2	46.5	72.3	64.9	38	67.8	35.9
CV2	0.29			1.1		
#3	24.5	80.5	48.5	61.6	65.7	48.7
CV3	0.57			4.15		

**Table 4:** Metabolic data for treated and non-treated subjects.

	Treated			Non-Treated		
Subject	#1	#2	#3	#1	#2	#3
**MAXIMAL TEST**						
VE (L·min^−1^)	154.1	78.8	152.4	146.5	93.5	132.9
VO2/Kg (mL·kg·min^−1^)	39.3	39.6	59	39.4	47.6	52.5
QR	1.127	1.064	1.13	1.121	1.016	1.087
VCO2 (L·min^−1^)	4.17	2.74	4.73	4.15	3.14	4.05
FC (lat·min^−1)^	159	177	173	152	183	171
Metabolic rate (kcal·day^−1^)	27,679	19,021	31,356	27,690	22,600	27,657
**SUBMAXIMAL TEST**						
1M-45	4947	4924	5218	5072	4663	5058
1M-45-O_2_	275.6	268.7	392.4	279.5	294.3	360.3
1M-45-QR	0.977	0.875	0.907	0.968	0.924	0.89
%VO_2_ max	77.1	74.2	77.1	79.9		
64.01M-45-kcal	641.3	429.8	726.8	666	445.5	645.8
Kcal·metres	0.130	0.087	0.139	0.131	0.096	0.128

## Data Availability

Not applicable.

## Supplementary Materials

The following are available online at https://www.mdpi.com/article/10.3390/ijerph18084128/s1, Figure S1: Impact of olive oil supplement on A. Leukocytes (109·L^−1^), B. Lactate (mmol·L^−1^), C. IL8 (pg·mL^−1^), D. IL6 (pg·mL^−1^) during and after intense exercise.
