# Supplementary material for: Impact of Olive Oil Supplement Intake on Dendritic Cell Maturation after Strenuous Physical Exercise: A Preliminary Study
Source: Int J Environ Res Public Health. 2021 Apr 14;18(8):4128. doi: 10.3390/ijerph18084128 (PMC8070678; doi:10.3390/ijerph18084128)
Supplement: Supplementary file 1 [file ijerph-18-04128-s001.pdf]

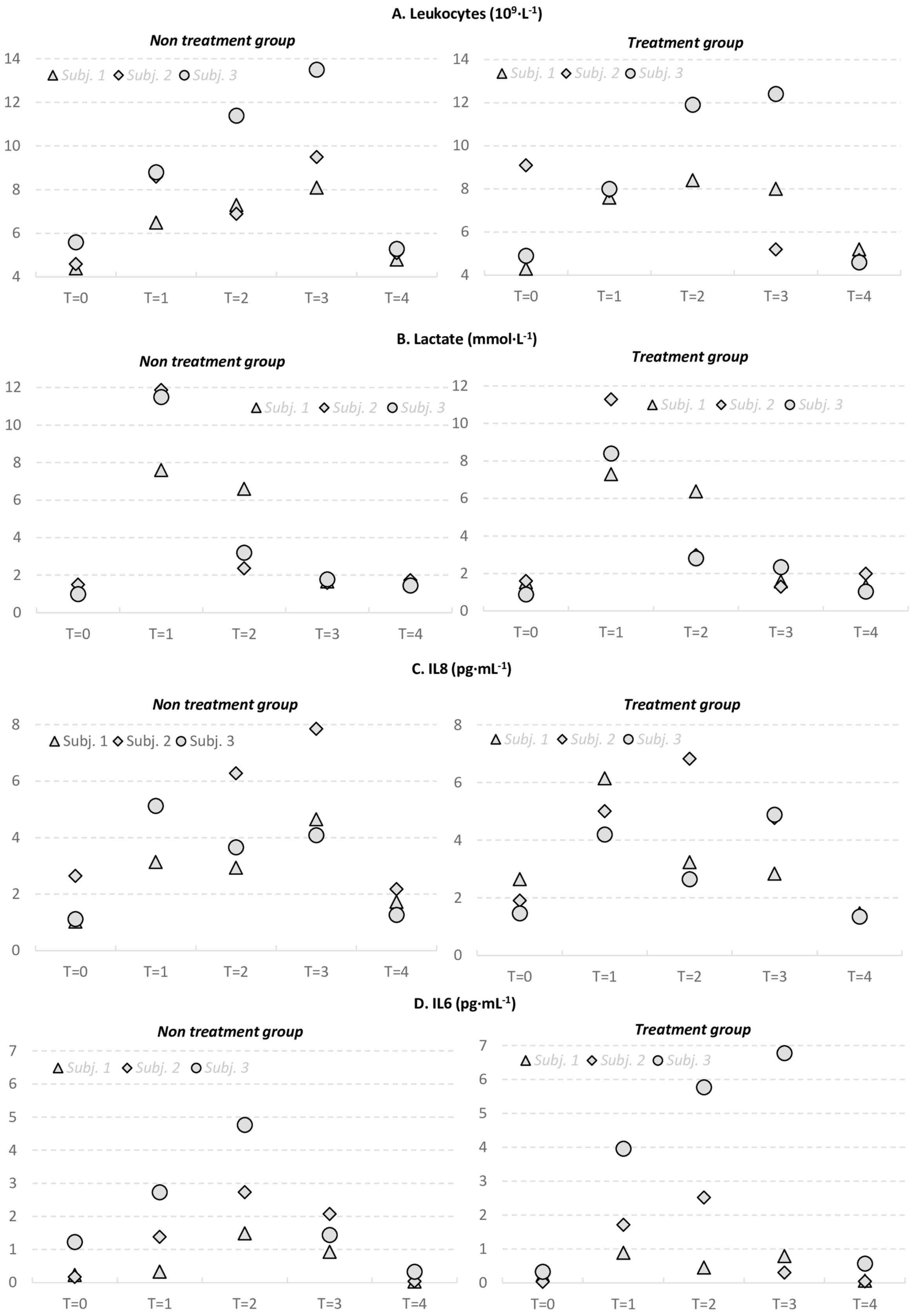

Figure S1. *Impact of olive oil supplement on* ***A****. Leukocytes ($10^9 \cdot L^{-1}$),* ***B.*** *Lactate ($mmol \cdot L^{-1}$),* ***C****. IL8 ($pg \cdot mL^{-1}$),* ***D.*** *IL6 ($pg \cdot mL^{-1}$) during and after intense exercise.*
